# Supplementary material for: Unveiling the Bioactive Potential of the Invasive Jellyfish Phyllorhiza punctata Through Integrative Transcriptomic and Proteomic Analyses
Source: Biomolecules. 2025 Aug 4;15(8):1121. doi: 10.3390/biom15081121 (PMC12383608; doi:10.3390/biom15081121)
Supplement: Supplementary file 1 [file biomolecules-15-01121-s001.zip › Suplementary Material/FileS3/PEPFOLD4/XP_065063848_5366/clusters.html]

 PEP-FOLD clusters


Download archive of all models  here   

|  |  |  |
| --- | --- | --- |
| # | file | sOPEP |
| # cluster 1 - Size: 4 |  |  |
|  | XP\_065063848\_5366-model1 | -11.6715 |
|  | XP\_065063848\_5366-model1.1 | -11.6715 |
|  | XP\_065063848\_5366-model1.2 | -10.9284 |
|  | XP\_065063848\_5366-model1.3 | -9.00657 |
| # cluster 2 - Size: 1 |  |  |
|  | XP\_065063848\_5366-model2 | -11.1004 |
| # cluster 3 - Size: 39 |  |  |
|  | XP\_065063848\_5366-model3 | -11.0723 |
|  | XP\_065063848\_5366-model3.1 | -11.0723 |
|  | XP\_065063848\_5366-model3.2 | -11.0723 |
|  | XP\_065063848\_5366-model3.3 | -11.0723 |
|  | XP\_065063848\_5366-model3.4 | -11.0723 |
|  | XP\_065063848\_5366-model3.5 | -11.0723 |
|  | XP\_065063848\_5366-model3.6 | -10.0639 |
|  | XP\_065063848\_5366-model3.7 | -10.0456 |
|  | XP\_065063848\_5366-model3.8 | -10.0456 |
|  | XP\_065063848\_5366-model3.9 | -10.0456 |
|  | XP\_065063848\_5366-model3.10 | -10.0456 |
|  | XP\_065063848\_5366-model3.11 | -10.0456 |
|  | XP\_065063848\_5366-model3.12 | -10.0456 |
|  | XP\_065063848\_5366-model3.13 | -10.0456 |
|  | XP\_065063848\_5366-model3.14 | -10.0456 |
|  | XP\_065063848\_5366-model3.15 | -10.0456 |
|  | XP\_065063848\_5366-model3.16 | -10.0456 |
|  | XP\_065063848\_5366-model3.17 | -10.0456 |
|  | XP\_065063848\_5366-model3.18 | -10.0456 |
|  | XP\_065063848\_5366-model3.19 | -10.0456 |
|  | XP\_065063848\_5366-model3.20 | -10.0456 |
|  | XP\_065063848\_5366-model3.21 | -9.46665 |
|  | XP\_065063848\_5366-model3.22 | -9.46665 |
|  | XP\_065063848\_5366-model3.23 | -9.42732 |
|  | XP\_065063848\_5366-model3.24 | -9.23808 |
|  | XP\_065063848\_5366-model3.25 | -9.1989 |
|  | XP\_065063848\_5366-model3.26 | -9.00575 |
|  | XP\_065063848\_5366-model3.27 | -8.76699 |
|  | XP\_065063848\_5366-model3.28 | -8.72279 |
|  | XP\_065063848\_5366-model3.29 | -8.72279 |
|  | XP\_065063848\_5366-model3.30 | -8.57613 |
|  | XP\_065063848\_5366-model3.31 | -8.37116 |
|  | XP\_065063848\_5366-model3.32 | -8.21929 |
|  | XP\_065063848\_5366-model3.33 | -8.20098 |
|  | XP\_065063848\_5366-model3.34 | -8.09855 |
|  | XP\_065063848\_5366-model3.35 | -8.07632 |
|  | XP\_065063848\_5366-model3.36 | -7.47437 |
|  | XP\_065063848\_5366-model3.37 | -7.47437 |
|  | XP\_065063848\_5366-model3.38 | -7.39481 |
| # cluster 4 - Size: 16 |  |  |
|  | XP\_065063848\_5366-model4 | -10.2373 |
|  | XP\_065063848\_5366-model4.1 | -10.2373 |
|  | XP\_065063848\_5366-model4.2 | -10.1812 |
|  | XP\_065063848\_5366-model4.3 | -10.1812 |
|  | XP\_065063848\_5366-model4.4 | -10.1812 |
|  | XP\_065063848\_5366-model4.5 | -9.92197 |
|  | XP\_065063848\_5366-model4.6 | -9.9014 |
|  | XP\_065063848\_5366-model4.7 | -9.86361 |
|  | XP\_065063848\_5366-model4.8 | -9.84515 |
|  | XP\_065063848\_5366-model4.9 | -9.84515 |
|  | XP\_065063848\_5366-model4.10 | -9.84134 |
|  | XP\_065063848\_5366-model4.11 | -9.84134 |
|  | XP\_065063848\_5366-model4.12 | -9.72429 |
|  | XP\_065063848\_5366-model4.13 | -9.72253 |
|  | XP\_065063848\_5366-model4.14 | -9.72253 |
|  | XP\_065063848\_5366-model4.15 | -9.69678 |
| # cluster 5 - Size: 4 |  |  |
|  | XP\_065063848\_5366-model5 | -9.85623 |
|  | XP\_065063848\_5366-model5.1 | -9.52093 |
|  | XP\_065063848\_5366-model5.2 | -9.52093 |
|  | XP\_065063848\_5366-model5.3 | -9.52093 |
| # cluster 6 - Size: 23 |  |  |
|  | XP\_065063848\_5366-model6 | -9.72865 |
|  | XP\_065063848\_5366-model6.1 | -9.72865 |
|  | XP\_065063848\_5366-model6.2 | -9.62836 |
|  | XP\_065063848\_5366-model6.3 | -9.51877 |
|  | XP\_065063848\_5366-model6.4 | -9.43022 |
|  | XP\_065063848\_5366-model6.5 | -9.38717 |
|  | XP\_065063848\_5366-model6.6 | -9.38717 |
|  | XP\_065063848\_5366-model6.7 | -9.38717 |
|  | XP\_065063848\_5366-model6.8 | -9.38717 |
|  | XP\_065063848\_5366-model6.9 | -9.38717 |
|  | XP\_065063848\_5366-model6.10 | -9.38717 |
|  | XP\_065063848\_5366-model6.11 | -9.38717 |
|  | XP\_065063848\_5366-model6.12 | -9.38717 |
|  | XP\_065063848\_5366-model6.13 | -9.38717 |
|  | XP\_065063848\_5366-model6.14 | -9.38717 |
|  | XP\_065063848\_5366-model6.15 | -9.38236 |
|  | XP\_065063848\_5366-model6.16 | -9.30961 |
|  | XP\_065063848\_5366-model6.17 | -9.24825 |
|  | XP\_065063848\_5366-model6.18 | -9.15946 |
|  | XP\_065063848\_5366-model6.19 | -9.15946 |
|  | XP\_065063848\_5366-model6.20 | -9.02289 |
|  | XP\_065063848\_5366-model6.21 | -8.36377 |
|  | XP\_065063848\_5366-model6.22 | -7.67636 |
| # cluster 7 - Size: 2 |  |  |
|  | XP\_065063848\_5366-model7 | -9.43329 |
|  | XP\_065063848\_5366-model7.1 | -9.36666 |
| # cluster 8 - Size: 3 |  |  |
|  | XP\_065063848\_5366-model8 | -9.02865 |
|  | XP\_065063848\_5366-model8.1 | -8.81044 |
|  | XP\_065063848\_5366-model8.2 | -8.73219 |
| # cluster 9 - Size: 5 |  |  |
|  | XP\_065063848\_5366-model9 | -8.73661 |
|  | XP\_065063848\_5366-model9.1 | -7.45091 |
|  | XP\_065063848\_5366-model9.2 | -7.45091 |
|  | XP\_065063848\_5366-model9.3 | -6.99812 |
|  | XP\_065063848\_5366-model9.4 | -6.35525 |
| # cluster 10 - Size: 1 |  |  |
|  | XP\_065063848\_5366-model10 | -8.30201 |
| # cluster 11 - Size: 1 |  |  |
|  | XP\_065063848\_5366-model11 | -6.73122 |
| # cluster 12 - Size: 1 |  |  |
|  | XP\_065063848\_5366-model12 | -5.36418 |
|  |  |  |
